# Infertility treatment and the growth of children from birth to 12 years of age: A national cohort study

**DOI:** 10.1371/journal.pone.0348091

**Published:** 2026-06-03

**Authors:** Yu-Shan Chang, Tsung Yu, Tzu-Jung Wong, Chung-Yi Li

**Affiliations:** 1 Department of Emergency Medicine, Chi Mei Medical Center, Tainan, Taiwan; 2 Department of Public Health, College of Medicine, National Cheng Kung University, Tainan, Taiwan; 3 Department of Healthcare Information and Management, School of Health and Medical Engineering, Ming Chuan University, Taoyuan, Taiwan; 4 Department of Public Health, College of Public Health, China Medical University, Taichung, Taiwan; 5 Department of Healthcare Administration, College of Medical and Health Science, Asia University, Taichung, Taiwan; University Hospital of Münster, GERMANY

## Abstract

**Background:**

Children conceived through infertility treatments may have altered birth outcomes and growth patterns compared to those conceived naturally. However, evidence on long-term growth trajectories, particularly distinguishing singletons and multiples, remains inconsistent. This study aimed to compare childhood growth trajectories from birth to 12 years of age between children conceived via infertility treatments and those conceived naturally, using a large national birth cohort.

**Methods:**

We analyzed the follow-up data (6 months to 12 years of age) from the Taiwan Birth Cohort Study, an ongoing, nationwide, longitudinal study of children. Our final sample included 20,507 singletons (20,306 naturally conceived [NC], 104 via intrauterine insemination [IUI], 97 via in vitro fertilization [IVF]) and 950 multiples (670 NC, 68 IUI, 212 IVF). We used linear mixed models with random coefficient cubic splines for age to assess the growth trajectories for weight, height, and body mass index (BMI) by infertility treatment status and logistic regression to analyze risk of rapid weight gain, overweight, obesity, and underweight.

**Results:**

Children conceived via IUI or IVF showed lower weight, height, and BMI in pooled analyses; however, these differences were largely attributable to the higher proportion of multiple births in the infertility treatment groups. After stratification by plurality, most differences were attenuated and not statistically significant, with the exception of slightly lower weight (−0.353 kg; p = 0.039) among IUI-conceived multiples. The risk of rapid weight gain (6 months–2 years) and underweight, overweight, or obesity (5.5–12 years) did not differ significantly by the conception mode after plurality was controlled.

**Conclusions:**

After accounting for plurality, children conceived through infertility treatments showed similar growth trajectories to those conceived naturally. These findings are reassuring for families using infertility treatments and highlight the importance of minimizing multiple births in assisted reproduction to support optimal child growth and health outcomes.

## Introduction

The Developmental Origins of Health and Disease (DOHaD) theory hypothesizes that early-life exposures influence health and disease risk later in life, with epigenetic changes potentially playing a key role in fetal programming mechanisms [1]. Low birth weight and preterm birth, both indicative of impaired intrauterine growth, along with rapid postnatal catch-up growth, are strong predictors of adverse cardiometabolic risk factors and chronic diseases in adulthood [2–4]. Therefore, understanding childhood growth trajectories is essential for assessing development and health across the life course.

Several studies have suggested that children conceived through medically assisted reproduction (MAR) have a shorter gestational age, lower birth weight, and a higher risk of being small for gestational age compared to those conceived naturally [5]. It has been hypothesized that these differences may result from factors such as ovarian stimulation, culture media, specific MAR procedures (e.g., intracytoplasmic sperm injection [ICSI] or frozen embryo transfer [FET]) [6–8], as well as the underlying causes of subfertility or infertility [9]. However, a systematic review by Bay et al. on the long-term growth of singletons found no significant differences in weight or height between children conceived through MAR and those conceived naturally [10].

In twin pregnancies, a recent systematic review by Marleen et al. found that MAR is associated with a higher risk of maternal complications but has a varied impact on perinatal outcomes compared to natural conception [11]. Furthermore, the association between MAR and growth patterns in twins remains unclear, with conflicting findings in the literature. For example, a Chinese cohort study of 26,818 twins reported no significant differences in growth from birth to 18 years between MAR-conceived and naturally conceived twins [12]. In contrast, a British cohort study of 13,528 twins suggested that MAR twins born after FET grew faster than those born after fresh embryo transfer or natural conception [13].

Given these inconsistencies, our study aimed to compare growth trajectories from birth to 12 years between children conceived via infertility treatments and those conceived naturally. We further stratified the analysis by singletons and multiples. This study was conducted using data from a long-term, population-based national birth cohort in Taiwan.

## Methods

### Study participants and design

The Taiwan Birth Cohort Study is an ongoing, nationwide, longitudinal study that recruited 21,648 children born in Taiwan in 2005. A two-stage stratified random sampling method was used to select a nationally representative sample. In the first stage, the study team selected 89 townships based on urbanization levels and total fertility rate. In the second stage, newborns were randomly selected from these townships using the probability proportional to size sampling method. Structured interviews were conducted with the primary caregivers, and baseline data were collected when the participants were 6 months old. Until 2009, follow-up surveys were conducted when the participants reached 1.5 years, 3 years, 5.5 years, 7 years, 8 years, 9 years, 12 years, and 13 years. More details can be found in previous publications [14,15].

Participant records were linked to the Birth Reporting Database, which provided information on plurality, birth weight, gestational age, and mode of delivery. The study cohort included 20,689 (95.6%) singletons and 959 (4.4%) multiples.

### Infertility treatment and covariates

At baseline, primary caregivers reported the method of conception using a standardized questionnaire, which included the following categories: in vitro fertilization (IVF), intrauterine insemination (IUI), or natural conception (NC). Among the participants, 311 (1.4%) were conceived via IVF, 172 (0.8%) via IUI, and 21,159 (97.7%) via NC. Additional covariates included child sex, child age, birth defects, breastfeeding status, pre-pregnancy body mass index (BMI), parental nationality, parental education, family monthly income, and parental smoking status.

### Anthropometric measurements

The Taiwanese government provides child preventive healthcare services, which include periodic health check-ups assessing growth and development. Healthcare providers conduct these assessments using standardized procedures. Primary caregivers were asked to record their child’s weight and length/height at the following ages: 1 month, 4 months, 6 months, 1 year, 1.5 years, 2 years, 2.5 years, 3 years, 4 years, 5 years, 5.5 years, 7 years, 8 years, 9.5 years, 12 years, and 13 years. For this study, we excluded measurements at 13 years of age. Weight was measured in kilograms (kg), and length/height in centimeters (cm). BMI was calculated as weight (kg) divided by height squared (m²).

### Statistical analysis

Not all children had complete data at every time point, and some had missing weight or height measurements. We excluded 6 participants with missing infertility treatment data and 15 participants with missing BMI values at all time points, resulting in a sample of 21,627 participants. We used linear mixed models with random coefficient cubic splines for age that adjusted for sex and plurality to predict weight and height at each time point for every participant. Missing values for weight or height were imputed using these predicted values.

Weight gain since birth was calculated at four time points (6 months, 1 year, 1.5 years, and 2 years) by determining the difference between weight at each time point and birth weight. We computed the mean and standard deviation (SD) of weight gain at each time point among all singletons. Standard deviation scores (SDS) were calculated for each child, and those with a score above 0.67 were classified as experiencing rapid weight gain [16].

BMI values were standardized into z-scores using the World Health Organization (WHO) Child Growth Standards [17] and Growth Reference Data for children aged 5–19 years [18]. A child was classified as obese if their BMI z-score exceeded 2, overweight if their BMI z-score exceeded 1 and underweight if their BMI z-score was below −2 [19]. The risk of obesity, overweight, and underweight was assessed at 5.5 years, 7 years, 8 years, 9.5 years, and 12 years.

Our final analysis sample included 21,457 participants, each with at least one BMI record between 6 months and 12 years of age. Separate analyses were conducted for singletons and multiples (including twin and higher-order multiple births). We used linear mixed models with random coefficient cubic splines for age to estimate mean differences in weight, height, and BMI from 6 months to 12 years (model-implied differences averaged over time), according to infertility treatment type (IUI, IVF, and NC). Additionally, logistic regression was used to estimate odds ratios for rapid weight gain at various time points as well as the risk of underweight, overweight, and obesity at different time points. All analyses were adjusted for potential confounders, including child sex, birth defects, breastfeeding, pre-pregnancy BMI, parental age, parental nationality, parental education, family income, and parental smoking status. Missing covariate data (all < 1% missing) were imputed using the most common response. Data analyses were conducted using STATA 15 (College Station, TX: StataCorp LLC). Statistical commands used to fit linear mixed models with random coefficient cubic splines were provided in the S1 Text.

### Ethical considerations

The Ethics Committees of the Health Promotion Administration, Ministry of Health and Welfare, Taiwan, approved the study protocol. All parents provided written informed consent before data collection. We accessed the data on June 1, 2024 and no authors had access to information that could identify individual participants.

## Results

### Characteristics of children

Infertility treatment was significantly more common among multiples (IUI: 7.2%; IVF: 22.3%) compared to singletons (IUI: 0.5%; IVF: 0.5%). On average, multiples had a shorter gestational age (35.6 weeks vs. 38.5 weeks) and lower birth weight (2,310 grams vs. 3,126 grams) than singletons. The baseline characteristics, stratified by plurality (singletons and multiples) and infertility treatment (NC, IUI, and IVF), are shown in Table 1.

**Table 1:** Baseline characteristics of the birth cohort by plurality and infertility treatment.

Characteristics	Singletons (n = 20507)						Multiples (n = 950)					
	NC (n = 20306)		IUI (n = 104)		IVF (n = 97)		NC (n = 670)		IUI (n = 68)		IVF (n = 212)	
	n	%	n	%	n	%	n	%	n	%	n	%
Child sex												
Boy	10634	52.4	61	58.7	55	56.7	366	54.6	35	51.5	124	58.5
Girl	9672	47.6	43	41.4	42	43.3	304	45.4	33	48.5	88	41.5
Birth defect	976	4.8	8	7.7	10	10.3	52	7.8	8	11.8	20	9.4
Breastfeeding	16694	82.2	89	85.6	87	89.7	517	77.2	61	89.7	174	82.1
Maternal nationality												
Native	17578	86.6	97	93.3	90	92.8	593	88.5	65	95.6	202	95.3
Foreign	2726	13.4	7	6.7	7	7.2	77	11.5	3	4.4	10	4.7
Maternal education												
Elementary school	818	4.0	2	1.9	0	0	19	2.8	0	0	2	0.9
Junior high school	2195	10.8	8	7.7	8	8.3	60	9.0	4	5.9	22	10.4
Senior high school	8148	40.1	27	26.0	32	33.0	278	41.5	24	35.3	57	26.9
College	4905	24.2	32	30.8	21	21.7	176	26.3	11	16.2	48	22.6
University	3511	17.3	31	29.8	28	28.9	116	17.3	22	32.4	67	31.6
Graduate school	694	3.4	4	3.9	8	8.3	20	3.0	6	8.8	15	7.1
Maternal smoking	1666	8.2	3	2.9	4	4.1	57	8.5	2	2.9	10	4.7
Paternal nationality												
Native	20036	98.7	104	100.0	95	97.9	655	97.8	66	97.1	211	99.5
Foreign	157	0.8	0	0	2	2.1	10	1.5	2	2.9	0	0
Paternal education												
Elementary school	291	1.4	0	0	0	0	14	2.1	2	2.9	2	0.9
Junior high school	2533	12.5	7	6.7	5	5.2	65	9.7	0	0	17	8.0
Senior high school	8091	39.9	30	28.9	24	24.7	249	37.2	28	41.2	59	27.8
College	4207	20.7	22	21.2	23	23.7	152	22.7	4	5.9	50	23.6
University	3530	17.4	30	28.9	30	30.9	143	21.3	19	27.9	47	22.2
Graduate school	1490	7.3	15	14.4	15	15.5	42	6.3	14	20.6	35	16.5
Paternal smoking	10980	54.1	39	37.5	35	36.1	357	53.3	23	33.8	83	39.2
Family monthly income												
<30000 TWD	2392	11.8	6	5.8	7	7.2	67	10.0	4	5.9	10	4.7
30000-50000 TWD	6138	30.2	19	18.3	19	19.6	206	30.8	2	2.9	37	17.5
50000-70000 TWD	5258	25.9	36	34.6	19	19.6	170	25.4	13	19.1	59	27.8
70000-100000 TWD	4230	20.8	22	21.2	21	21.7	143	21.3	27	39.7	46	21.7
100000-150000 TWD	1611	7.9	18	17.3	22	22.7	66	9.9	14	20.6	42	19.8
150000-200000 TWD	334	1.6	2	1.9	4	4.1	15	2.2	2	2.9	8	3.8
≥200000 TWD	274	1.4	1	1.0	4	4.1	0	0	6	8.8	10	4.7
	**Mean**	**SD**	**Mean**	**SD**	**Mean**	**SD**	**Mean**	**SD**	**Mean**	**SD**	**Mean**	**SD**
Maternal age	29.3	4.8	33.1	4.1	34.6	4.2	29.7	4.4	33.2	3.6	33.6	4.0
Paternal age	33.2	5.6	35.8	4.6	37.3	4.8	33.3	5.3	36.3	4.3	36.8	5.0
Pre-pregnancy BMI	21.0	3.2	22.1	4.1	21.6	2.7	21.0	3.1	20.8	3.0	21.7	3.8
Gestational age (wk)	38.5	1.5	38.4	1.5	38.1	1.9	35.8	2.2	35.6	2.0	35.3	2.5
Birth weight (g)	3126.6	426.2	3080.6	458.7	3114.1	470.2	2325.0	450.8	2253.6	380.4	2280.7	520.7

**Abbreviations:** BMI, body mass index; IUI, intrauterine insemination; IVF, in vitro fertilization; NC, natural conception; SD, standard deviation; TWD, New Taiwan dollar.

Within both singleton and multiple births, infertility treatment (IUI or IVF) was associated with a higher proportion of male infants and a higher prevalence of birth defects compared with NC. Parents who conceived via infertility treatment were also older, had higher educational attainment and family income, and were more likely to be native-born. In addition, breastfeeding was more common among children conceived through infertility treatment, while maternal and paternal smoking rates were lower. Pre-pregnancy BMI was slightly higher in the infertility treatment groups.

### Child growth at all assessment points

**Fig 1:**
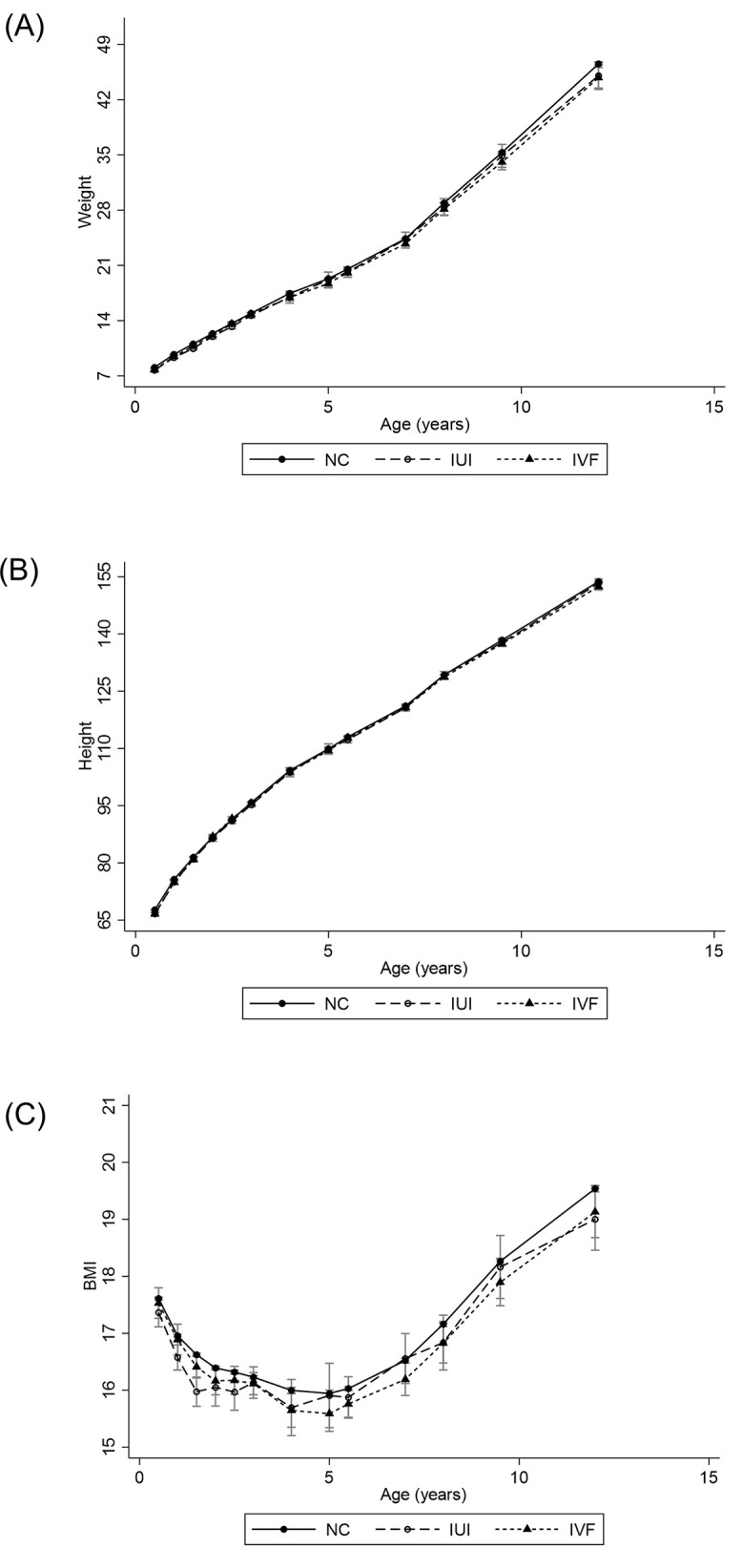
(A) Trajectory of mean weight in children conceived through intrauterine insemination (IUI), in vitro fertilization (IVF), and natural conception (NC) from 6 months to 12 years of age; (B) Trajectory of mean height in children conceived through IUI, IVF, and NC from 6 months to 12 years of age; (C) Trajectory of mean body mass index (BMI) in children conceived through IUI, IVF, and NC from 6 months to 12 years of age.

Among all children (singletons and multiples), infertility treatment (IUI or IVF) was associated with lower weight, shorter height, and lower BMI from 6 months to 12 years compared to the NC group (see Fig 1 and Table 2). These differences were largely driven by the higher proportion of multiples in the infertility treatment groups. After stratification by plurality, the differences in weight, height, and BMI were substantially attenuated, and most associations became statistically non-significant.

**Table 2:** Adjusted mean differences in growth from 6 months to 12 years of age by infertility treatment.

Outcomes	All children (n = 21457)			Singletons (n = 20507)			Multiples (n = 950)		
	Estimate	95% CI	P-value	Estimate	95% CI	P-value	Estimate	95% CI	P-value
Weight (kg)									
IUI vs NC	−0.349	−0.543 to −0.154	<0.001	−0.232	−0.480 to 0.016	0.067	−0.353	−0.689 to −0.018	0.039
IVF vs NC	−0.207	−0.354 to −0.060	0.006	−0.061	−0.321 to 0.200	0.647	−0.008	−0.223 to 0.207	0.940
Height (cm)									
IUI vs NC	−0.838	−1.246 to −0.429	<0.001	−0.423	−0.945 to 0.099	0.112	−0.604	−1.318 to 0.111	0.098
IVF vs NC	−0.557	−0.866 to −0.247	<0.001	−0.088	−0.634 to 0.458	0.751	0.329	−0.132 to 0.790	0.161
BMI									
IUI vs NC	−0.256	−0.453 to −0.059	0.011	−0.185	−0.435 to 0.066	0.149	−0.203	−0.572 to 0.165	0.279
IVF vs NC	−0.112	−0.261 to 0.037	0.139	0.049	−0.213 to 0.311	0.713	−0.044	−0.281 to 0.193	0.717

**Abbreviations:** BMI, body mass index; CI, confidence interval; IUI, intrauterine insemination; IVF, in vitro fertilization; NC, natural conception.

Models were adjusted for child sex, birth defects, breastfeeding, pre-pregnancy BMI, parental age, parental nationality, parental education, family income, and parental smoking status.

Among singletons, the adjusted mean differences in growth from 6 months to 12 years for children conceived via IUI compared with NC were −0.232 kg for weight (p = 0.067), −0.423 cm for height (p = 0.112), and −0.185 units for BMI (p = 0.149). Corresponding differences for IVF versus NC were −0.061 kg for weight (p = 0.647), −0.088 cm for height (p = 0.751), and 0.049 units for BMI (p = 0.713).

Among multiples, children conceived via IUI weighed significantly less than those conceived naturally (−0.353 kg, p = 0.039), while differences in height (−0.604 cm, p = 0.098) and BMI (−0.203 units, p = 0.279) were not statistically significant. No significant differences were observed among multiples conceived via IVF compared with NC for weight (−0.008 kg, p = 0.940), height (0.329 cm, p = 0.161), or BMI (−0.044 units, p = 0.717).

### Prevalence of rapid weight gain from 6 months to 2 years

The prevalence of rapid weight gain from 6 months to 2 years, stratified by plurality and infertility treatment, is presented in Table 3. Overall, multiples had a higher risk of rapid weight gain compared to singletons (28.3% vs. 21.4% at 2 years). After stratifying by plurality, no significant differences were found in the risk of rapid weight gain between infertility treatment groups and the NC group. For example, at 2 years, the risk of rapid weight gain in the IUI group was slightly lower than in the NC group among singletons (adjusted odds ratio [aOR]: 0.685, p = 0.156; Table 4). Conversely, the IVF group had a slightly higher risk compared to the NC group (aOR: 1.250, p = 0.355). However, neither estimate was statistically significant.

**Table 3:** Comparison of outcome prevalence in singletons and multiples according to infertility treatment.

Outcomes and Age	Singletons (n = 20507)						Multiples (n = 950)					
	NC (n = 20306)		IUI (n = 104)		IVF (n = 97)		NC (n = 670)		IUI (n = 68)		IVF (n = 212)	
	n	%	n	%	n	%	n	%	n	%	n	%
*Rapid weight gain*												
6 months	4698	23.1	19	18.3	22	22.7	257	38.4	17	25.0	81	38.2
1 year	4458	22.0	20	19.2	24	24.7	262	39.1	21	30.9	87	41.0
1.5 years	4564	22.5	13	12.5	19	19.6	210	31.3	21	30.9	72	34.0
2 years	4352	21.4	18	17.3	24	24.7	188	28.1	19	27.9	62	29.3
*Underweight*												
5.5 years	864	4.3	1	1.0	1	1.0	36	5.4	5	7.4	9	4.3
7 years	661	3.3	1	1.0	3	3.1	37	5.5	2	2.9	8	3.8
8 years	571	2.8	4	3.9	4	4.1	30	4.5	2	2.9	8	3.8
9.5 years	540	2.7	2	1.9	3	3.1	24	3.6	0	0	8	3.8
12 years	680	3.4	4	3.9	2	2.1	25	3.7	5	7.4	9	4.3
*Overweight*												
5.5 years	5614	27.7	33	31.7	32	33.0	145	21.6	8	11.8	47	22.2
7 years	6634	32.7	41	39.4	34	35.1	178	26.6	14	20.6	51	24.1
8 years	7370	36.3	42	40.4	40	41.2	191	28.5	15	22.1	54	25.5
9.5 years	8130	40.0	49	47.1	46	47.4	227	33.9	19	27.9	66	31.1
12 years	6807	33.5	41	39.4	36	37.1	171	25.5	14	20.6	59	27.8
*Obesity*												
5.5 years	2295	11.3	10	9.6	10	10.3	43	6.4	2	2.9	16	7.6
7 years	2948	14.5	19	18.3	16	16.5	56	8.4	5	7.4	19	9.0
8 years	3325	16.4	19	18.3	18	18.6	71	10.6	7	10.3	23	10.9
9.5 years	3694	18.2	21	20.2	21	21.7	84	12.5	6	8.8	32	15.1
12 years	2526	12.4	10	9.6	12	12.4	46	6.9	5	7.4	18	8.5

**Abbreviations:** IUI, intrauterine insemination; IVF, in vitro fertilization; NC, natural conception

**Table 4:** Adjusted odds ratios for risk of rapid weight gain from 6 months to 2 years of age by infertility treatment.

Age	Singletons (n = 20507)			Multiples (n = 950)		
	Estimate	95% CI	P-value	Estimate	95% CI	P-value
6 months						
IUI vs NC	0.704	0.423 to 1.172	0.177	0.579	0.309 to 1.085	0.088
IVF vs NC	0.987	0.605 to 1.609	0.958	1.106	0.764 to 1.600	0.595
1 year						
IUI vs NC	0.782	0.475 to 1.288	0.335	0.654	0.363 to 1.180	0.159
IVF vs NC	1.152	0.718 to 1.849	0.558	1.137	0.791 to 1.636	0.487
1.5 years						
IUI vs NC	0.437	0.242 to 0.789	0.006	0.861	0.472 to 1.571	0.625
IVF vs NC	0.831	0.498 to 1.385	0.477	1.032	0.706 to 1.509	0.871
2 years						
IUI vs NC	0.685	0.406 to 1.155	0.156	0.960	0.523 to 1.760	0.894
IVF vs NC	1.250	0.779 to 2.004	0.355	0.946	0.643 to 1.393	0.780

**Abbreviations:** CI, confidence interval; IUI, intrauterine insemination; IVF, in vitro fertilization; NC, natural conception.

Models were adjusted for child sex, birth defects, breastfeeding, pre-pregnancy BMI, parental age, parental nationality, parental education, family income, and parental smoking status.

### Prevalence of underweight, overweight, and obesity from 5.5 to 12 years

The prevalence of underweight, overweight, and obesity from 5.5 to 12 years, stratified by plurality and infertility treatment, is presented in Table 3. Overall, multiples had a higher risk of underweight (4.1% vs. 3.4% at 12 years) and a lower risk of overweight (25.7% vs. 33.6%) and obesity (7.3% vs. 12.4%) compared to singletons at the same age. After stratifying by plurality, no significant differences were found in the risk of underweight, overweight, or obesity between infertility treatment groups and the NC group. Adjusted risks for different outcomes at various time points are provided in Table 5. For example, at 12 years, the risk of obesity in the IUI group was slightly lower than in the NC group among singletons (aOR: 0.643, p = 0.209), while the risk in the IVF group was slightly higher (aOR: 1.159, p = 0.644). However, neither estimate was statistically significant.

**Table 5:** Adjusted odds ratios for underweight, overweight and obesity from 5.5 years to 12 years of age by infertility treatment.

Outcomes and Age	Singletons (n = 20507)			Multiples (n = 950)		
	Estimate	95% CI	P-value	Estimate	95% CI	P-value
*Underweight*						
5.5 years						
IUI vs NC	0.258	0.036 to 1.856	0.178	2.227	0.729 to 6.806	0.160
IVF vs NC	0.263	0.036 to 1.895	0.185	1.009	0.438 to 2.324	0.983
7 years						
IUI vs NC	0.294	0.041 to 2.117	0.224	0.292	0.035 to 2.432	0.255
IVF vs NC	0.929	0.291 to 2.962	0.901	0.790	0.320 to 1.953	0.610
8 years						
IUI vs NC	1.522	0.553 to 4.190	0.416	0.775	0.159 to 3.774	0.752
IVF vs NC	1.672	0.605 to 4.617	0.321	1.082	0.437 to 2.682	0.864
9.5 years						
IUI vs NC	0.723	0.176 to 2.967	0.652	Not estimable		
IVF vs NC	1.235	0.386 to 3.952	0.722	1.107	0.442 to 2.773	0.828
12 years						
IUI vs NC	1.137	0.412 to 3.140	0.804	1.241	0.402 to 3.831	0.707
IVF vs NC	0.571	0.139 to 2.341	0.436	1.260	0.511 to 3.105	0.616
*Overweight*						
5.5 years						
IUI vs NC	1.137	0.740 to 1.747	0.558	0.580	0.256 to 1.314	0.191
IVF vs NC	1.378	0.893 to 2.125	0.147	1.021	0.665 to 1.568	0.924
7 years						
IUI vs NC	1.345	0.890 to 2.033	0.159	1.165	0.593 to 2.291	0.658
IVF vs NC	1.260	0.820 to 1.937	0.292	0.965	0.638 to 1.460	0.867
8 years						
IUI vs NC	1.127	0.747 to 1.702	0.569	0.874	0.453 to 1.687	0.688
IVF vs NC	1.327	0.874 to 2.015	0.184	0.804	0.537 to 1.201	0.287
9.5 years						
IUI vs NC	1.259	0.836 to 1.897	0.270	1.017	0.548 to 1.886	0.958
IVF vs NC	1.464	0.967 to 2.218	0.072	0.910	0.618 to 1.338	0.631
12 years						
IUI vs NC	1.194	0.782 to 1.824	0.411	1.132	0.565 to 2.265	0.727
IVF vs NC	1.262	0.819 to 1.943	0.291	1.171	0.772 to 1.774	0.458
*Obesity*						
5.5 years						
IUI vs NC	0.740	0.377 to 1.454	0.382	0.995	0.210 to 4.723	0.995
IVF vs NC	0.999	0.512 to 1.950	0.998	1.685	0.821 to 3.459	0.155
7 years						
IUI vs NC	1.253	0.741 to 2.118	0.400	1.972	0.650 to 5.984	0.230
IVF vs NC	1.346	0.774 to 2.341	0.293	1.621	0.836 to 3.144	0.153
8 years						
IUI vs NC	1.043	0.615 to 1.769	0.875	1.761	0.698 to 4.442	0.231
IVF vs NC	1.324	0.778 to 2.254	0.302	1.234	0.683 to 2.231	0.486
9.5 years						
IUI vs NC	1.016	0.606 to 1.704	0.951	0.956	0.369 to 2.476	0.925
IVF vs NC	1.406	0.845 to 2.339	0.189	1.270	0.756 to 2.133	0.366
12 years						
IUI vs NC	0.643	0.322 to 1.281	0.209	1.807	0.598 to 5.461	0.294
IVF vs NC	1.159	0.619 to 2.173	0.644	1.553	0.776 to 3.109	0.214

**Abbreviations:** CI, confidence interval; IUI, intrauterine insemination; IVF, in vitro fertilization; NC, natural conception.

Models were adjusted for child sex, birth defects, breastfeeding, pre-pregnancy BMI, parental age, parental nationality, parental education, family income, and parental smoking status.

## Discussion

We identified several key findings. First, although children conceived via IUI or IVF appeared to have lower weight, shorter height, and lower BMI than those conceived naturally when all children were analyzed together, these differences were primarily attributable to the higher proportion of multiple births in the IUI and IVF groups. Second, after stratifying by plurality, no significant differences in weight, height, or BMI were observed between the infertility treatment groups and the NC group. Among multiples, children in the IUI group might have slightly lower weight than those in the NC group. Third, the risks of rapid weight gain (6 months–2 years) and underweight, overweight, and obesity (5.5–12 years) did not significantly differ by infertility treatment status in either singletons or multiples. Overall, after accounting for plurality, we found no significant differences in growth trajectories between children conceived through infertility treatments and those conceived naturally.

The DOHaD hypothesis suggests that prenatal and perinatal exposure to environmental factors—such as undernutrition, stress, or environmental chemicals—can alter an individual’s phenotype and increase the risk of non-communicable diseases in adulthood [20]. Concerns have been raised that some MAR procedures may directly affect embryos, potentially impairing fetal and childhood growth. For example, IVF exposes gametes and embryos to various hormones, culture media, and in vitro manipulation techniques, including prolonged embryo culture, cryopreservation and thawing, prenatal genetic testing, and embryo biopsy [21]. Laboratory evidence indicates that these procedures can induce molecular changes, alter epigenetics and gene expression, and may influence placental and fetal development [22]. Therefore, monitoring the long-term health and growth of children conceived via infertility treatments is essential.

Most of our data showed that after accounting for plurality, infertility treatments were not significantly associated with childhood growth trajectories, suggesting that the higher rate of multiples, rather than the infertility treatments themselves, was the key factor related to impaired child growth. These findings demonstrate that the influence of MAR on long-term offspring growth is negligible if the rate of multiple births is controlled. Our findings also highlight the importance of minimizing multiple pregnancies in MAR. Singleton pregnancies result in better maternal and child outcomes than multiple pregnancies [23]. Therefore, most professional reproductive medicine societies, including those in Taiwan, advocate for elective single embryo transfer in IVF to reduce pregnancy risks associated with multiple births. As a result, the rate of multiples following IVF is declining globally [24,25].

In our analysis stratified by plurality, we found that multiples conceived via IUI had significantly lower weight than those conceived naturally. This finding aligns with research by Yeung et al., who observed that twins conceived via ovulation induction (with or without IUI) were smaller from birth to three years of age than naturally conceived twins [16]. Ovarian stimulation for IUI commonly involves clomiphene citrate, letrozole, or injectable gonadotropins, with gonadotropin use significantly increasing the risk of multiple gestation [26]. Ovarian hyperstimulation may lead to ovulation of immature oocytes or alter endometrial function due to exposure to high hormone levels (e.g., estradiol and progesterone), resulting in impaired placental function and restricted fetal growth [16]. Supporting this hypothesis, studies have shown that frozen embryo transfers—where endometrial preparation is more natural—carry a lower risk of small-for-gestational-age infants compared to fresh embryo transfers [27].

The major strengths of this study include its large, nationally representative cohort from Taiwan (n = 21,457), reducing selection bias. Participants were followed from 6 months of age, with repeated weight and height measurements enabling longitudinal analysis. Many previous studies lacked sufficient sample sizes or long-term follow-up, making our study a valuable addition to the literature. However, the numbers of children conceived via infertility treatment among singletons were relatively small. As a result, non-significant findings in singleton analyses should be interpreted with caution, as they do not necessarily indicate equivalence but may reflect limited statistical power and wide confidence intervals.

Study limitations should be considered. Infertility treatment status was self-reported rather than sourced from medical records, and child growth data were reported by caregivers rather than directly measured by healthcare professionals, introducing potential measurement errors, which might have biased the results towards the null. Additionally, children conceived by infertility treatments had shorter gestational ages and lower birth weights than naturally conceived children, even after stratifying by plurality (see **Table 1**). We did not adjust for these variables, as they are intermediate factors in the causal pathway. The observed lower weight in multiples conceived via IUI may partially reflect these differences.

Furthermore, although we adjusted for several sociodemographic and perinatal factors, information on maternal and paternal alcohol consumption and maternal nutrition during pregnancy was not available in the datasets accessed for this study and therefore could not be considered. In addition, breastfeeding was assessed only as a binary variable, as data on duration or exclusivity were not available. Finally, detailed information on the underlying etiology or pathophysiology of infertility among parents who underwent infertility treatment was not captured in the available data. These unmeasured factors may contribute to residual confounding and warrant further investigation in future studies with more detailed clinical and behavioral information.

Our findings contribute to the literature on long-term growth outcomes among children conceived through infertility treatments. Consistent with prior studies, we observed no clear evidence of differences in weight, height, or BMI after accounting for plurality, suggesting that infertility treatment itself is unlikely to have a clinically relevant impact on childhood growth. Instead, the higher prevalence of multiple births largely explains the differences observed in un-stratified analyses. This finding reassures couples undergoing infertility treatments. However, we lacked detailed MAR-related data, such as whether ICSI was used or whether embryo transfer was fresh or frozen. Future efforts should focus on establishing national MAR registries and birth cohort studies to assess the long-term effects of evolving MAR techniques on child growth and health outcomes. Importantly, our findings emphasize the need to minimize multiple births following MAR to optimize pregnancy and child health outcomes.

## Supporting information

S1 Text Supplementary Materials. STATA commands used to fit linear mixed models.
(DOCX)

## Abbreviations

BMI: Body mass index
CI: Confidence interval
DOHaD: Developmental Origins of Health and Disease
FET: Frozen embryo transfer
ICSI: Intracytoplasmic sperm injection
IUI: Intrauterine insemination
IVF: In vitro fertilization
MAR: Medically assisted reproduction
NC: Natural conception
OR: Odds ratio
SD: Standard deviation
SDS: Standard deviation scores
