# Supplementary material for: Infertility treatment and the growth of children from birth to 12 years of age: A national cohort study
Source: PLoS One. 2026 Jun 3;21(6):e0348091. doi: 10.1371/journal.pone.0348091 (PMC13232800; doi:10.1371/journal.pone.0348091)
Supplement: S1 Text — STATA commands used to fit linear mixed models. (DOCX) [file pone.0348091.s001.docx]

**Supplementary Materials**

Knots were determined based on the empirical age distribution, follow-up density and growth dynamics. The following STATA commands were used for analysis of all children.

*mkspline agespw = age, cubic knots(1 4 5 7 8)*

*mkspline agesph = age, cubic knots(1 1.5 2 4 7 8)*

*mkspline agespb = age, cubic knots(1 1.5 2 3 4 5 5.5 7 8 9.5)*

*xtmixed wt_ i.art2 i.sex i.e3q i.bfeed bmi_m mage_6m fage_6m i.nationality_m i.nationality_f i.edu_m i.edu_f i.income i.smoke_m i.smoke_f agespw* || newid: age, cov(uns)*

*xtmixed ht_ i.art2 i.sex i.e3q i.bfeed bmi_m mage_6m fage_6m i.nationality_m i.nationality_f i.edu_m i.edu_f i.income i.smoke_m i.smoke_f agesph* || newid: age, cov(uns)*

*xtmixed bmi i.art2 i.sex i.e3q i.bfeed bmi_m mage_6m fage_6m i.nationality_m i.nationality_f i.edu_m i.edu_f i.income i.smoke_m i.smoke_f agespb* || newid: age, cov(uns)*
